# Supplementary material for: Laboratory validation of a clinical metagenomic next-generation sequencing assay for respiratory virus detection and discovery
Source: Nat Commun. 2024 Nov 12;15:9016. doi: 10.1038/s41467-024-51470-y (PMC11558004; doi:10.1038/s41467-024-51470-y)
Supplement: Supplementary file 1 — Supplementary Information [file 41467_2024_51470_MOESM1_ESM.pdf]

SUPPLEMENTARY INFORMATION

Supplementary Table 1. Evaluation and interpretation of human background based on detection of MS2 phage and/or viral reads.

Result*	Interpretation
MS2 phage DETECTED; viral pathogen DETECTED	Results are valid.
MS2 phage NOT DETECTED; viral pathogen DETECTED	Results are valid.
MS2 phage DETECTED; viral pathogen NOT DETECTED	Results are valid.
MS2 phage NOT DETECTED; viral pathogen NOT DETECTED	Results are invalid.

*All possible outcomes regarding the validity of mNGS testing based on detection of MS2 and/or a viral pathogen in the clinical sample.

Supplementary Table 2. Evaluation of human background based on ERCC RNA spike-in quantifications results.

IC RPM Ratio Range*	Interpretive Comment
>5	Due to very low host background, estimated limits of detection in this clinical sample are approximately 1 log lower than the stated limits of detection for this test.
0.5 – 5	None
0.05 – 0.5	Due to moderate host background, estimated limits of detection in this clinical sample are approximately 1 log higher than the stated limits of detection for this test.
0.005 – 0.05	Due to high host background, estimated limits of detection in this clinical sample are approximately 2 logs higher than the stated limits of detection for this test.
0 – 0.005	Due to very high host background, estimated limits of detection in this clinical sample are approximately 3 logs higher than the stated limits of detection for this test.

$* \ IC\ RPM\ ratio\ = \frac{ERCC\ RPM\ sample}{ERCC\ RPM\ NC}, for\ RPM\ NC\ > 0, or\ 1\ for\ RPM\ NC\ =\ 0$. Abbreviations: ERCC, External RNA Controls Consortium; IC, internal control; NC, negative control; RPM, reads per million.

Supplementary Table 3. Definitions of clinical severity for respiratory viral infections (related to Figure 2B).

Asymptomatic	No symptoms from respiratory viral infection, regardless of whether hospitalized or not hospitalized
Mild	All cases of respiratory viral infection not requiring supplemental oxygen OR if hospitalized, mild upper respiratory symptoms only, including fever, sore throat, cough, rhinorrhea, loss of sense of smell or taste.
Moderate	Hospitalized due to aan cute respiratory viral infection AND/OR any oxygen support delivered by low-flow nasal cannula (defined as ≤6L for adults) AND/OR any systemic, non-respiratory symptom attributed to the acute respiratory infection<br>(e.g., neonatal fever, dehydration, new diagnosis of diabetes, appendicitis, necrosis of extremities, diarrhea, encephalopathy, renal insufficiency, coagulation abnormalities, etc.)
Severe	Must be hospitalized due to an acute respiratory viral infection with high-flow oxygen requirement (defined as >6L delivered by high-flow nasal cannula for adults, BIPAP (bilevel positive airway pressure), intubation with mechanical ventilation, or ECMO (extracorporeal membrane oxygenation) AND/OR evidence of end-organ failure (acute renal failure requiring dialysis, coagulation abnormalities resulting in bleeding or stroke, diabetic ketoacidosis, hemodynamic instability requiring vasopressors). These are nearly always patients in the ICU.

Supplementary Table 4. Assessment of exclusivity by testing of a mixture of 10 non-viral organisms (bacteria and fungi) from a commercially available microbial community standard (related to Table 1).

		ZymoBIOMICS™ Standard Replicate 1 (RPM ratio)	ZymoBIOMICS™ Standard Replicate 2 (RPM ratio)	ZymoBIOMICS™ Standard Replicate 3 (RPM ratio)
RNA Viral Pathogen		Not detected	Not detected	Not detected
Bacteria	*Pseudomonas aeruginosa*	109,961	100,759	100,425
	Escherichia coli	2,775	2,911	2,886
	Salmonella enterica	249,809	264,668	260,984
	Lactobacillus fermentum	366,693	364,440	366,732
	Enterococcus faecalis	240,805	247,572	248,073
	Staphylococcus aureus	3,097	3,011	3,069
	Listeria monocytogenes	150,586	150,851	151,388
	Bacillus subtilis	2.11	2.16	2.14
Fungi	*Saccharomyces cerevisiae*	210,062	205,745	208,848
	Cryptococcus neoformans	309,698	291,633	296,407
Internal Spiked RNA Viral Control	*Escherichia* phage MS2	0.20	0.09	0.10

Reads per million (RPM) ratios for bacteria, fungi, and an internal spiked *Escherichia coli* MS2 RNA phage control detected in three replicate samples of the ZymoBIOMICS™ Microbial Community Standard (Zymo Research). The RPM ratio is calculated as RPM_{sample} / RPM_{NTC}, where NTC is the non-template control.

Supplementary Table 5. Interference study results for mucoid BAL samples (n=14) (related to Table 1).

sample ID*	MS2 Phage count	ERCC		Preprocessed Reads	Respiratory Virus Detected by mNGS			Respiratory Virus Detected by PCR	Valid run*	Final result concordance
		RPM ratio	Interpretation		count	RPM	Virus species			
BAL122	0	0.00013	Very high host background	26,294,115			Not Detected	Flu A	no	Excluded
BAL124	0	0.00002	Very high host background	7,636,468			Not Detected	RSV A	no	Excluded
BAL127	0	0.00003	Very high host background	28,672,379			Not Detected	Flu A	no	Excluded
BAL137	0	0.00003	Very high host background	11,915,589			Not Detected	Flu A	no	Excluded
BAL148	77	0.00701	High host background	21,449,028	18	0.8	Rhinovirus A	Rhinovirus / Enterovirus	yes	Concordant
BAL157	39	0.0031	Very high host background	33,527,383	1	0	Rhinovirus A (subthreshold)	Rhinovirus / Enterovirus	yes	Concordant
BAL170	0	0.00002	Very high host background	18,272,481	1	0.1	Rhinovirus A (subthreshold)	Rhinovirus / Enterovirus	yes	Concordant
BAL202	1	0.00021	Very high host background	19,331,651	26	1.3	Rhinovirus A	Rhinovirus / Enterovirus	yes	Concordant
BAL223	0	0.00037	Very high host background	23,275,778	1	0	Rhinovirus A (subthreshold)	Rhinovirus / Enterovirus	yes	Concordant
BAL238	0	0.00004	Very high host background	13,261,601	6	0.5	Rhinovirus A	Rhinovirus / Enterovirus	yes	Concordant
BAL241	0	0	Very high host background	6,189,715	2	0.3	Rhinovirus A (subthreshold)	Rhinovirus / Enterovirus	yes	Concordant
BAL248	0	0	Very high host background	4,322,243			Not Detected	Rhinovirus / Enterovirus	no	Excluded
BAL257	0	0.00034	Very high host background	21,640,525	25	1.2	Rhinovirus A	Rhinovirus / Enterovirus	yes	Concordant
BAL288	0	0.00002	Very high host background	7,372,716			Not Detected	Rhinovirus / Enterovirus	no	Excluded

*Analysis of 14 PCR-positive bronchoalveolar lavage samples to evaluate the potential matrix effect from samples with high host background; invalid runs, with neither detection of spiked MS2 phage nor a respiratory virus, were excluded from the analysis. Abbreviations: ERCC, External RNA Controls Consortium; ST, subthreshold; BAL, bronchoalveolar lavage; RPM, reads per million.

Supplementary Table 6. Discrepant mNGS Positive Results Compared to Original Respiratory Viral Panel Clinical Testing (n = 14 Samples, related to Figure 4).

Organisms Detected by Clinical RVP	Organisms Detected by mNGS	Results of Discrepancy Testing	Additional Data	Final Result of mNGS by DTCA
RSV	RSV<br>Rhinovirus B	(+) Rhinovirus		**TP** RSV<br>**TP** Rhinovirus
RSV	RSV<br>EV-D68	(+) Enterovirus		**TP** RSV<br>**TP** Enterovirus
RSV	RSV<br>Adenovirus C	(+) Adenovirus		**TP** RSV<br>**TP** Adenovirus
Adenovirus<br>Metapneumovirus	Adenovirus C<br>Metapneumovirus<br>Bocavirus	Bocavirus ND	Bocavirus mNGS reads are specific to the viral genus. Patient with clinical syndrome consistent with acute vi infection.	**TP** Adenovirus<br>**TP** Metapneumovirus<br>**TP** Bocavirus
ND	Coronavirus OC43	Coronavirus OC43 ND	Detected by mNGS x 2 with high RPM and high coverage. No evidence of cross-contamination. Patient with clinical syndrome consistent with acute respiratory viral infection.	**TP** Coronavirus OC43
ND	Coronavirus OC43	(+) Coronavirus OC43		**TP** Coronavirus OC43
ND	Rhinovirus A	(+) Rhinovirus		**TP** Rhinovirus
ND	Rhinovirus A	(+) Rhinovirus		**TP** Rhinovirus
ND	Coronavirus HKU1	Coronavirus HKU1 ND		**FP** Coronavirus HKU1
ND	Coronavirus HKU1	(+) Coronavirus HKU1		**TP** Coronavirus HKU1
ND	Parainfluenza 1<br>SARS-CoV-2	(+) Parainfluenza 1<br>SARS-CoV-2 ND		**TP** Parainfluenza 1<br>**FP** SARS-CoV-2
ND	Coronavirus NL63	(+) Coronavirus NL63		**TP** Coronavirus NL63
ND	Rhinovirus C	Rhinovirus ND	Mismatches in the forward primer and probe sequences for rhinovirus/enterovirus PCR. No evidence of contamination. Patient with clinical syndrome consistent with acute respiratory viral infection.	**TP** Rhinovirus
ND	Coronavirus OC43	NT		**NA** Coronavirus OC43

Final mNGS result after discrepancy testing and clincial adjudication are shown in boldfaced text. False positive results were only considered if the organism was included as a target for RVP. Each result was assessed independently, in case of multiple positive results (multiple organisms), the corresponding TP/FP call was divided by the number of organisms detected within each sample (e.g. 1 TP + 1 FP in a same sample = ½ TP + ½ FP). This allowed to keep the observation constant (see Methods for further details). Abbreviations: RVP, respiratory viral panel; DTCA, discrepancy testing and clinical adjudication; RSV, respiratory syncytial virus; EV-D68, enterovirus D68; RPM, reads per million; ND, not detected; NT, not tested; TP, true positive; FP, false positive; NA, not available.

Supplementary Table 7. Discrepant mNGS Negative Results Compared to Original Respiratory viral Panel Clinical Testing (n = 9 Samples, related to Figure 4).

Organisms Detected By Clinical RVP	CT Value or MFI from Clinical RVP	Organisms Detected by mNGS	High Host Background?	Results of Discrepancy Testing	Discrepancy Testing Platform	Final Result of mNGS by DTCA
Influenza A<br>Parainfluenza 3	NA	Influenza A	Y	(+) Influenza A<br>Parainfluenza 3 ND	MagPix	**TP** Influenza A<br>**TN** Parainfluenza 3
Influenza A and SARS-CoV-2	SARS-CoV-2 CT: 36.4	Influenza A	Y	Influenza A NT<br>SARS-CoV-2 ND	Verigene	**TP** Influenza A<br>**TN** SARS-CoV2
Influenza A<br>RSV	NA	ND	Y	Influenza A ND<br>(+) RSV B	GenMark	**TN** Influenza A<br>**FN** RSV
Influenza A and SARS-CoV-2	NA	SARS-CoV-2	N	Influenza A ND<br>SARS-CoV-2 NT	MagPix	**TP** SARS-CoV-2<br>**TN** Influenza A
Rhinovirus/Enterovirus	Rhinovirus/Enterovirus MFI: 52	ND	Y	Rhinovirus/Enterovirus ND	MagPix	**TN** Rhinovirus/Enterovirus
Rhinovirus/Enterovirus	NA	ND	N	Rhinovirus/Enterovirus ND	MagPix	**TN** Rhinovirus/Enterovirus
Rhinovirus/Enterovirus<br>Parainfluenza 2	NA	Rhinovirus A	N	(+) Rhinovirus/Enterovirus<br>Parainfluenza 2 ND	MagPix	**TP** Rhinovirus/Enterovirus<br>**TN** Parainfluenza 2
Metapneumovirus	NA	ND	Y	(+) Metapneumovirus	GenMark	**FN** Metapneumovirus
RSV A	NA	ND	Y	RSV ND	MagPix	**TN** RSV A

Final mNGS result after discrepancy testing and clincial adjudication are shown in boldfaced text. Abbreviations: RVP, respiratory viral panel; MFI, mean fluorescence units; DTCA, discrepancy testing and clinical adjudication; RSV, respiratory syncytial virus; ND, not detected; Y, yes; N, no; NA, not available; NT, not tested; TP, true positive; FN, false negative; MAGPIX, Luminex MagPix® system for RVP testing; GenMark, Genmark ePlex® Respiratory Pathogen Panel 2 for RVP testing; Verigene, Verigene® Respiratory Pathogen Flex Test for RVP testing.

Supplementary Table 8. Costs for the UCSF viral respiratory mNGS assay (related to Figure 1). Section titles and cost subtotals and totals are shown in boldfaced text. The total cost per test is shown with a light blue background.

**Name of Test**	**UCSF viral respiratory mNGS Assay**	
No. specimens per run (3 runs/week)	11	
Total # of patient tests performed annually	1,716	
No. Controls per run (positive and negative controls)	2	
**Total samples per run (Patient & Controls)**	13	
**Reagents and Supplies**	**Price/unit**	**Reagent cost/test**
KingFisher extraction with DNase treatment (500 tests/unit)	3,604.80	38.86
Tecan MagicPrep DNA Seq kits (32 tests/unit)	1,550.00	48.44
NextSeq Mid Output (13 tests/unit)	1,178.10	90.62
**Subtotal reagent & supply costs**		177.92
% adjustment for consumables	20%	
**Total + % adjustment for consumables**		213.50
**Supply cost per run**		2,312.97
**Supply cost per patient sample**		**210.27**
Annual Service Cost: Thermo Fisher KingFisher extraction $7000, Tecan MagicPrep $6,000.00, NextSeq $35,293.50	53,649.50	
**Service cost per test**		31.26
Instrument purchase price (if purchased) Analysis server $60,000, Data storage server $30,000, Thero Fisher KingFisher $64,578.65	154,578.65	
**Instrument cost per test**		18.02
**Total cost of reagents/supplies per test**		**259.55**
Labor time		minutes
Prepare/clean work areas -both pre & post PCR		15.00
extraction (automated)		30.00
cDNA_DNA_protocol		60.00
MagicPrep preperation - hands on		15.00
MagicPrep library (automated)		0.00
Qubit & NextSeq loading		60.00
Bioinformatics (SURPI+, server time)		15.00
Post analysis Reporting Sunquest		5.00
**Labor costs per run (mins)**		200.00
**Labor costs per sample (mins)**		18.18
Daily maintenance (mins)		15.00
**Total labor cost per test**		**33.18**
**Total cost per test**		**292.73**
