# Supplementary material for: Laboratory validation of a clinical metagenomic next-generation sequencing assay for respiratory virus detection and discovery
Source: Nat Commun. 2024 Nov 12;15:9016. doi: 10.1038/s41467-024-51470-y (PMC11558004; doi:10.1038/s41467-024-51470-y)
Supplement: Supplementary file 3 — Description of Additional Supplementary Files [file 41467_2024_51470_MOESM3_ESM.pdf]

Description of Additional Supplementary Files

File Name: Supplementary Data 1
Description: Clinical and laboratory metadata associated with the patients whose respiratory samples were analyzed in this study.
