# Supplementary material for: Laboratory validation of a clinical metagenomic next-generation sequencing assay for respiratory virus detection and discovery
Source: Nat Commun. 2024 Nov 12;15:9016. doi: 10.1038/s41467-024-51470-y (PMC11558004; doi:10.1038/s41467-024-51470-y)
Supplement: Supplementary file 5 — Reporting Summary [file 41467_2024_51470_MOESM5_ESM.pdf]

nature portfolio

Corresponding author(s): Charles Chiu

Last updated by author(s): Jul 26, 2024

# Reporting Summary

Nature Portfolio wishes to improve the reproducibility of the work that we publish. This form provides structure for consistency and transparency in reporting. For further information on Nature Portfolio policies, see our Editorial Policies and the Editorial Policy Checklist.

## Statistics

For all statistical analyses, confirm that the following items are present in the figure legend, table legend, main text, or Methods section.

| n/a | Confirmed | |
|---|---|---|
| ☐ | ☒ | The exact sample size (*n*) for each experimental group/condition, given as a discrete number and unit of measurement |
| ☐ | ☒ | A statement on whether measurements were taken from distinct samples or whether the same sample was measured repeatedly |
| ☐ | ☒ | The statistical test(s) used AND whether they are one- or two-sided<br>*Only common tests should be described solely by name; describe more complex techniques in the Methods section.* |
| ☐ | ☒ | A description of all covariates tested |
| ☐ | ☒ | A description of any assumptions or corrections, such as tests of normality and adjustment for multiple comparisons |
| ☐ | ☒ | A full description of the statistical parameters including central tendency (e.g. means) or other basic estimates (e.g. regression coefficient) AND variation (e.g. standard deviation) or associated estimates of uncertainty (e.g. confidence intervals) |
| ☐ | ☒ | For null hypothesis testing, the test statistic (e.g. *F*, *t*, *r*) with confidence intervals, effect sizes, degrees of freedom and *P* value noted<br>*Give P values as exact values whenever suitable.* |
| ☒ | ☐ | For Bayesian analysis, information on the choice of priors and Markov chain Monte Carlo settings |
| ☒ | ☐ | For hierarchical and complex designs, identification of the appropriate level for tests and full reporting of outcomes |
| ☒ | ☐ | Estimates of effect sizes (e.g. Cohen's *d*, Pearson's *r*), indicating how they were calculated |

*Our web collection on statistics for biologists contains articles on many of the points above.*

## Software and code

Policy information about availability of computer code

Data collection

MNucleic acid extraction
500 µL of upper respiratory swab or BAL fluid was centrifuged at 16,000 x g for 10 minutes. The MagMAX™ Viral/Pathogen II (MVP II) Nucleic Acid Isolation Kit (cat # A48383, Thermo Fisher Scientific, Waltham, MA) and the KingFisher™ Flex Purification System with a 96 deep-well head (Thermo Fisher Scientific, Waltham, MA) were used for total nucleic acid extraction. This protocol was modified to include DNase treatment using TURBO™ DNase (cat # AM2238, Thermo Fisher Scientific, Waltham, MA) as a host depletion step during extraction. Bacteriophage MS2 (cat # 22-156-880, Zeptometrix, Buffalo, NY) was added to all samples including the negative control as an internal qualitative control.

Library preparation and sequencing
Simultaneous reverse transcription of purified RNA, spiked in with ERCC RNA controls (cat # 4456740, Invitrogen, Waltham, MA), and ribosomal RNA (rRNA) depletion were carried out using NEBNext® Ultra™ II RNA First Strand Synthesis Module (cat #s E7771S/ E7771L, New England Biolabs, Ipswich, MA) and QIAseq FastSelect-rRNA HMR Kit (cat # 334385, Qiagen, Germantown, MD), respectively, followed by second strand cDNA synthesis using Sequenase™ Version 2.0 DNA Polymerase (cat # 70775Z1000UN, Thermo Fisher Scientific, Waltham, MA). Complementary DNA (cDNA) was purified using AMPure XP beads (cat # A63881, Beckman Coulter, Brea, CA) and loaded on the MagicPrep NGS instrument (Tecan Genomics, Inc., Männedorf, Switzerland) to undergo end-repair, adapter ligation and barcoding, amplification (25 cycles) and purification using the DNA-Seq Mech kit (cat #s 30186627/30186628/30186629, Tecan Genomics, Inc., Männedorf, Switzerland). Libraries were quantified and normalized using the Qubit dsDNA HS Assay (cat # Q32854, Thermo Fisher Scientific, Waltham, MA) on the Qubit Flex (Thermo Fisher Scientific, Waltham, MA). Final pooled libraries were sequenced as single-end reads on either the Illumina (San Diego, CA) MiniSeq using the Rapid Reagent Kit (100 cycles) or on the Illumina NextSeq 550 using the Mid-Output or High-Output Kit (150 cycles).

Data analysis

The SURPI+ computational pipeline, run as a container (v1.0.0) on either a secure server or cloud infrastructure, was used for identification of respiratory viral pathogens from mNGS data. Reads were preprocessed by trimming of adapters and removal of low-complexity and low-quality sequences, followed by computational subtraction of human reads. The Scalable Nucleotide Alignment Program (SNAP) nucleotide aligner was run using an edit distance of 16 against the National Center for Biotechnology Information (NCBI) nucleotide (NT) database (March 2019, with inclusion of the SARS-CoV-2 WuHan-Hu-1 genome accession number NC_045512) , which was pre-filtered to retain only viral reads. The pipeline was modified to include "tagging", or annotation, of entries from reference sequences that constitute a subset of the NCBI NT database, such as FDA-ARGOS. Note that the FDA-ARGOS database, while quality controlled and regulated, contains only 1,428 microbial strains, the majority of which are bacterial. It had also not been updated with recent viruses such as SARS-CoV-2; thus, this study did not detect any reads matching to viral genomes in FDA-ARGOS. The pipeline was modified to accommodate additional reference databases as needed such as GISAID. The pipeline was also modified to use SPAdes (v3.15.4) and DIAMOND (v2.0.15), respectively, for optional de novo assembly of reads into contiguous sequences (contigs) and translated nucleotide sequence alignment for identification of sequence-divergent viruses. Viral reads were identified using DIAMOND at a e-value cutoff of 10^-5. Coverage maps were automatically generated by mapping SURPI+-classified viral reads to the most likely reference genome.

Quality control metrics for the assay were based on those previously established for cerebrospinal fluid, and include a minimum of 5 million preprocessed reads per sample, >75% of data with quality score >30 (Q>30), and successful detection of the 4 respiratory viruses in the PC and the internal spiked MS2 phage control. A criterion of ≥3 non-overlapping viral reads or contigs aligning to the target viral genome was considered a positive detection.

Statistical analyses were performed using scipy (version 1.5.3) and rstatix (version 0.7.0) packages as implemented in Python (version 3.7.12) and R (version 4.0.3), respectively. The non-parametric Mann-Whitney U test was used for pairwise comparisons of viral load medians, while the Kruskal-Wallis H test was used for comparisons of medians across all severity groups. Probit regression analyses were done using scipy (version 1.5.3), numpy (version 1.19.1) and statsmodels (version 0.12.2) as implemented in Python software (version 3.7.12).

For manuscripts utilizing custom algorithms or software that are central to the research but not yet described in published literature, software must be made available to editors and reviewers. We strongly encourage code deposition in a community repository (e.g. GitHub). See the Nature Portfolio guidelines for submitting code & software for further information.

## Data

Policy information about availability of data

All manuscripts must include a data availability statement. This statement should provide the following information, where applicable:

- Accession codes, unique identifiers, or web links for publicly available datasets
- A description of any restrictions on data availability
- For clinical datasets or third party data, please ensure that the statement adheres to our policy

Human-subtracted raw sequence data were submitted to the Sequence Read Archive (SRA) database. (BioProject accession number PRJNA1084017 and umbrella BioProject accession number PRJNA171119). Source data are provided as a Source Data file. Sequence metadata is available in a Zenodo data repository (https://zenodo.org/doi/10.5281/zenodo.10553378).

Custom scripts and code for data analyses and visualization are available in a Zenodo data repository (https://zenodo.org/doi/10.5281/zenodo.10553378 ). The SURPI+ bioinformatics pipeline is described in prior publications (Naccache, et al., Genome Research, 2014, 24(7):1180-1192; Miller, et al., Genome Research, 2019, 29(5):831-842). The code for SURPI+ includes proprietary algorithms for taxonomic classification, filtering, and pathogen software that have been filed under US patent 11380421, "Pathogen detection using next generation sequencing". Pleae contact the University of California Office of Technology Management regarding access to and use of the software.

## Research involving human participants, their data, or biological material

Policy information about studies with human participants or human data. See also policy information about sex, gender (identity/presentation), and sexual orientation and race, ethnicity and racism.

Reporting on sex and gender

Sex and gender were not considered in study design. Analysis was performed on aggregated data.

Reporting on race, ethnicity, or other socially relevant groupings

Race, ethnicity or any socially relevant groupings were not considered in study design. Analysis was performed on aggregated data.

Population characteristics

To evaluate accuracy, 191 residual samples after routine clinical testing were obtained from the UCSF Clinical Microbiology Laboratory, including 110 virus-positive samples (104 upper respiratory swab samples and 6 BAL fluids) from patients with acute respiratory infection along with 81 virus-negative samples (52 upper respiratory swab samples and 29 BAL fluids)

Recruitment

Residual laboratory-confirmed virus-positive upper respiratory swab or BAL samples from clinical patient testing were retrieved from the UCSF Clinical Microbiology Laboratory. Acceptable upper respiratory swab samples included (1) bilateral nasopharyngeal swabs, (2) bilateral anterior nares swabs, (3) oropharyngeal swabs, (4) combined nasopharyngeal and oropharyngeal swabs, and (5) combined oropharyngeal/mid-turbinate nasal swabs. All samples were required to meet minimal sample handling, storage, and volume requirements for inclusion in our study. Samples were stored at 4°C for <24 hr before being de-identified, aliquoted, and stored in -80°C freezer prior to mNGS processing, thus undergoing one freeze-thaw cycle. All samples meeting minimal volume (450 uL), sample handling (at most one freeze-thaw step), and storage (kept frozen at -80°C) requirements were included in this study.

| | |
|---|---|
| Ethics oversight | Samples along with clinical and laboratory metadata were collected according to a biobanking protocol with waiver of consent approved by the UCSF Institutional Review Board (protocol no. 11-05519) |

Note that full information on the approval of the study protocol must also be provided in the manuscript.

# Field-specific reporting

Please select the one below that is the best fit for your research. If you are not sure, read the appropriate sections before making your selection.

☒ Life sciences ☐ Behavioural & social sciences ☐ Ecological, evolutionary & environmental sciences

For a reference copy of the document with all sections, see nature.com/documents/nr-reporting-summary-flat.pdf

# Life sciences study design

All studies must disclose on these points even when the disclosure is negative.

| | |
|---|---|
| Sample size | No sample size was calculated. All samples meeting minimal volume (450 uL), sample handling (at most one freeze-thaw step), and storage (kept frozen at -80°C) requirements were included in this study. |
| Data exclusions | No data were excluded from the analysis. |
| Replication | We measured intra-assay precision by testing two PC and two NC samples within the same run using different barcodes across 20 runs and inter-assay precision by testing 20 PC and 20 NC samples using different barcodes across 20 separate runs. Essential agreement (EA) was 100% and intra- and inter-assay precision were within our a priori established limits of <10% and <30% log-transformed coefficients of variation in reads per million, respectively. |
| Randomization | Not applicable. |
| Blinding | Researchers were blinded during processing as all samples were assigned a random unique ID. |

# Reporting for specific materials, systems and methods

We require information from authors about some types of materials, experimental systems and methods used in many studies. Here, indicate whether each material, system or method listed is relevant to your study. If you are not sure if a list item applies to your research, read the appropriate section before selecting a response.

## Materials & experimental systems

| n/a | Involved in the study | |
|---|---|---|
| ☒ | ☐ | Antibodies |
| ☒ | ☐ | Eukaryotic cell lines |
| ☒ | ☐ | Palaeontology and archaeology |
| ☒ | ☐ | Animals and other organisms |
| ☒ | ☐ | Clinical data |
| ☒ | ☐ | Dual use research of concern |
| ☒ | ☐ | Plants |

## Methods

| n/a | Involved in the study | |
|---|---|---|
| ☒ | ☐ | ChIP-seq |
| ☒ | ☐ | Flow cytometry |
| ☒ | ☐ | MRI-based neuroimaging |

## Plants

| | |
|---|---|
| Seed stocks | Not applicable. |
| Novel plant genotypes | Not applicable. |
| Authentication | Not applicable. |
